# Leisure-time physical activity improves happiness, health, and mood profile better than work-related physical activity

**DOI:** 10.1371/journal.pone.0307744

**Published:** 2024-07-24

**Authors:** Albertas Skurvydas, Natalja Istomina, Ruta Dadeliene, Daiva Majauskiene, Emilija Strazdaite, Ausra Lisinskiene, Dovile Valanciene, Aiste Barbora Uspuriene, Asta Sarkauskiene

**Affiliations:** 1 Department of Rehabilitation, Physical and Sports Medicine, Faculty of Medicine, Institute of Health Sciences, Vilnius University, Vilnius, Lithuania; 2 Institute of Health Sciences, Faculty of Medicine, Vilnius University, Vilnius, Lithuania; 3 Education Academy, Vytautas Magnus University, Kaunas, Lithuania; 4 Faculty of Law, Vilnius University, Vilnius, Lithuania; 5 Department of Sports, Recreation and Tourism, Klaipeda University, Klaipeda, Lithuania; University of Tartu, ESTONIA

## Abstract

**Background:**

In an online survey of Lithuanian adults (n = 1140) aged 18 to 64 years, we sought to better understand the factors influencing the structure of physical activity (PA). We hypothesised that the PA paradox (i.e. the benefits of PA will be much greater during leisure-time than work-related or household moderate to vigorous PA) occurs more subjectively for psychological well-being indicators, than physiological well-being indicators, and should not depend on age or gender.

**Methods:**

An online questionnaire was distributed as to potential participants through the *Facebook* social networking website within the period May 2021 to December 2021. PA was assessed using the long version of the *International Physical Activity Questionnaire (IPAQ)*. Mood responses were assessed using the *Brunel Mood Scale (BRUMS-LTU)*. Emotional intelligence was assessed using the *Schutte Self-Report Emotional Intelligence Test (SSREIT)*. Perceived stress was assessed using the *10-item Perceived Stress Scale (PSS-10*). Descriptive analysis, a two-way analysis of variance, and linear regression analysis were used to interpret the data.

**Results:**

The results revealed that a PA paradox occurred in women and men in terms of health, happiness, vigour and perceived stress, and only in women according to morbidity and overeating. According to the regression analysis, women’s and men’s subjective health (β = 0.135; p < 0.001), happiness (β = 0.084; p = 0.018) and vigour (β = 0.169; p < 0.001) were significantly positively, and perceived stress (β = -0.088; p = 0.009) negatively correlated (regardless of age) only with leisure-time moderate to vigorous PA. ‘Healthy’, i.e. the amount of leisure-time PA in men decreases with age, while it does not change in women.

**Conclusions:**

We believe that this study has expanded a clearer understanding of the PA paradox and its possible application to improving the health of individuals of different age groups.

## Introduction

Physical activity (PA) undoubtedly combats many chronic diseases, prolongs a person’s life span, improves physical and mental performance [1–11]. It has been clearly shown that mental health status is linked to both PA levels and to sedentary behaviour independent of PA [1]. Furthermore, mood is one of the indicators of psychological well-being and mental health [11]. No one doubts that PA leads not only to better health, but also to a complete diet, abstemiousness [12], good rest and especially sleep [13], shorter periods of sedentary behaviour [1, 5, 14], emotion regulation and impulse control [6, 15, 16] and many other factors (diet, smoking, alcohol consumption, cognitive leisure activity and meditation) affect human health and well-being [17]. For example, Tapia-Serrano et al. distinguished the three most important factors for healthy behaviour of schoolchildren, i.e. PA, screen time, and sleep duration [13]. Also, there is data that increasing the volume of PA can lead to higher levels of happiness and enhanced subjective well-being, regardless of the intensity of PA [18, 19].

Although the latest World Health Organization (2020) guidelines on PA and sedentary behaviour [5] clearly show that any kind of PA is beneficial for human health, researchers have recently revealed a PA paradox, which is manifested in the fact that leisure-time PA is more beneficial for health than work-related PA [20–27]. This PA paradox was manifested in the study of many indicators, such as mortality of all causes [20, 21, 28], cardiovascular disease mortality [26], long-term sickness absence [20], longevity [29] and systolic blood pressure (SBP) [25]. This paradox has been found to occur differently in men and women [22, 29]. For example, a meta-analysis showed that higher levels of occupational PA in men increased the risk of early mortality of all causes by 18% compared with those who engaged in lower levels of occupational PA [22]. However, there was no such interaction among women; there was even a reverse trend. Research in Norway, conducted with women and men, showed that moderate to high occupational PA contributes to longevity in men, but occupational PA does not improve longevity in women [29]. However, there is still no unequivocal opinion regarding the ‘physical activity paradox’ [22, 23], as it may depend on the type of professions and the amount and intensity of PA during occupational PA [27, 30]. We did not find any studies that comprehensively examined whether this paradox manifests itself, and if it occurs, does it depend on gender and age for health-related and well-being indicators (e.g. health, vigour, happiness, morbidity, blood pressure), healthy behaviour indicators (sleeping time, body mass index (BMI), overeating). Furthermore, we did not find works on the possible (if at all possible) associations between the PA paradox and emotional intelligence (EI) and perceived stress and effectiveness of logical thinking. Our previous studies showed that there is a significant positive correlation between PA and EI, but there is no such correlation between logical thinking and PA [6, 7, 31, 32]. However, in those studies, we only measured overall moderate to vigorous PA (MVPA) and did not differentiate between work-related and leisure-time PA. Based on the research cited above, we hypothesise that the PA paradox (i.e. the benefits of PA will be much greater during leisure-time MVPA (MVPAlt) than work-related MVPA (MVPAw) or household MVPA (MVPAh)), if it occurs at all, occurs more subjectively for psychological well-being indicators (e.g. health, vigour, happiness), than physiological well-being indicators (e.g. body mass index (BMI), systolic blood pressure (SBP), morbidity and sleeping time), and should not depend on age or gender. Additionally, we raise another hypothesis that if leisure-time PA brings more benefits to psychological well-being and health, then it should be chosen more by people with higher EI and better logical (rational) thinking, i.e. there should be a positive correlation between leisure-time PA and EI and effectiveness of logical thinking, and there should be a negative correlation between perceived stress and leisure-time PA.

The primary goal of our study was to evaluate the hypothesis that leisure-time PA promotes happiness, health, and mood profile more effectively than work-related physical activity across diverse age groups, encompassing both women and men.

## Material and methods

### Survey design and procedure

A total of 1140 individuals participated in the study, comprising 831 who identified as women (72.9%) and 309 who identified as men (27.1%). The participants ranged in age from 18 to 64 years (women age = 41.9 ± 11.6 yr; men age = 40.1 ± 11.2 yr). An online questionnaire created with *Google Forms* was distributed as to potential participants through the *Facebook* social networking website within the period May 2021 to February 2022. Participants were informed of the purpose of the research, which received ethical approval from the Human Research Ethics Committee at Klaipėda University. Written consent for participation was obtained from each respondent. All research participants were informed that the information provided in the anonymous survey will be used for research purposes.

### Instruments

PA was assessed using the long version of the ***International Physical Activity Questionnaire*** (*IPAQ*). The *IPAQ* is a 27-item self-reported measure of PA, comprising of four activity domains: work-related PA, transportation PA, domestic PA and recreational PA. *IPAQ* items assess the frequency of PA for each domain by assessing the number of days per week during which the subject engages in PA and the average duration of PA, described in hours and minutes. The total weekly PA was estimated weighting the time spent performing each activity intensity with its metabolic equivalent (MET) energy expenditure. The METs of vigorous, moderate and low intensity activities were 8.0, 4.0 and 3.3 METs, respectively [33].

Mood responses were assessed using the Lithuanian-language version of the ***Brunel Mood Scale*** *(BRUMS-LTU)*, consisting of 24 items designed to assess tension, depression, anger, vigour, fatigue and confusion. In this case, we used only the Vigour subscale, whose items were energetic, active, lively, alert. Participants responded on a five-point Likert scale of *0 = not at all*, *1 = a little*, *2 = moderately*, *3 = quite a bit* and *4 = extremely*, with total possible subscale scores ranging from 0–16 [34, 35].

Emotional intelligence was assessed using the ***Schutte Self-Report Emotional Intelligence Test*** (*SSREIT*). The *SSREIT* is 33-item questionnaire divided into four subscales, which are: Perception of emotions (10 items), Ability to deal with one’s own emotions (9 items), Ability to deal with the emotions of others (8 items) and Use of emotions (5 items). The items are designed to be answered on a five-point scale ranging from *1 = strongly disagree* to *5 = strongly agree*. Total scores ranged from 33 to 165, with higher scores indicating greater ability in the area of EI [36].

Perceived stress was assessed using the ***10-item Perceived Stress Scale*** *(PSS-10)*. The *PSS-10* is a 10-item self-reported questionnaire designed to assess the extent to which the individual has perceived situations in their life as unpredictable, uncontrollable and overloading over the past month. It consists of 10 questions, which are designed to be answered on a five-point scale ranging from *0 = never* to *4 = very often*. Scores for the four positively stated items (4, 5, 7, 8) are reversed. Total scores ranged from 0 to 40, with higher scores indicating a higher perceived stress level [37].

Logical thinking was assessed using the ***Cognitive Reflection Test*** *(CRT)*. The *CRT* is a three-item measure of reflective reasoning, i.e., the tendency to suppress an intuitive (incorrect) response in favour of a more conscious (correct) response. The test consists of three questions and is scored as the total number of correct answers [38].

Subjective health was assessed through the ***Subjective Health Self-Assessment***. For this purpose, the following four-point scale was used: *1 = poor health*, *2 = satisfactory health*, *3 = good health* and *4 = excellent health*. Education level was assessed according to education indicators of adult education level.

Other parameters such as participants’ BMI (we calculated this indicator based on the height and weight values given by the respondents), overeating, sleeping time, SBP, happiness, and morbidity were also evaluated.

### Data analysis

Descriptive statistics and normality tests for the continuous data were performed. Data were analysed by using IBM SPSS Statistics software (*version 22; IBM Corp*., *Armonk*, *NY*, *USA*). We used descriptive analysis, two-way analysis of variance (ANOVA) and linear regression analysis. For all tests, statistical significance was defined as p < 0.05. The reliability of mean differences was evaluated using the t-test criterion and the p-value of independent samples. If significant effects were found in ANOVA, Tukey’s post hoc adjustment was used for multiple comparisons within each repeated-measures ANOVA. The reliability of the questionnaires was calculated as the Cronbach’s alpha coefficient. The parameter β was estimated as the regression coefficient.

## Results

**Fig 1:**
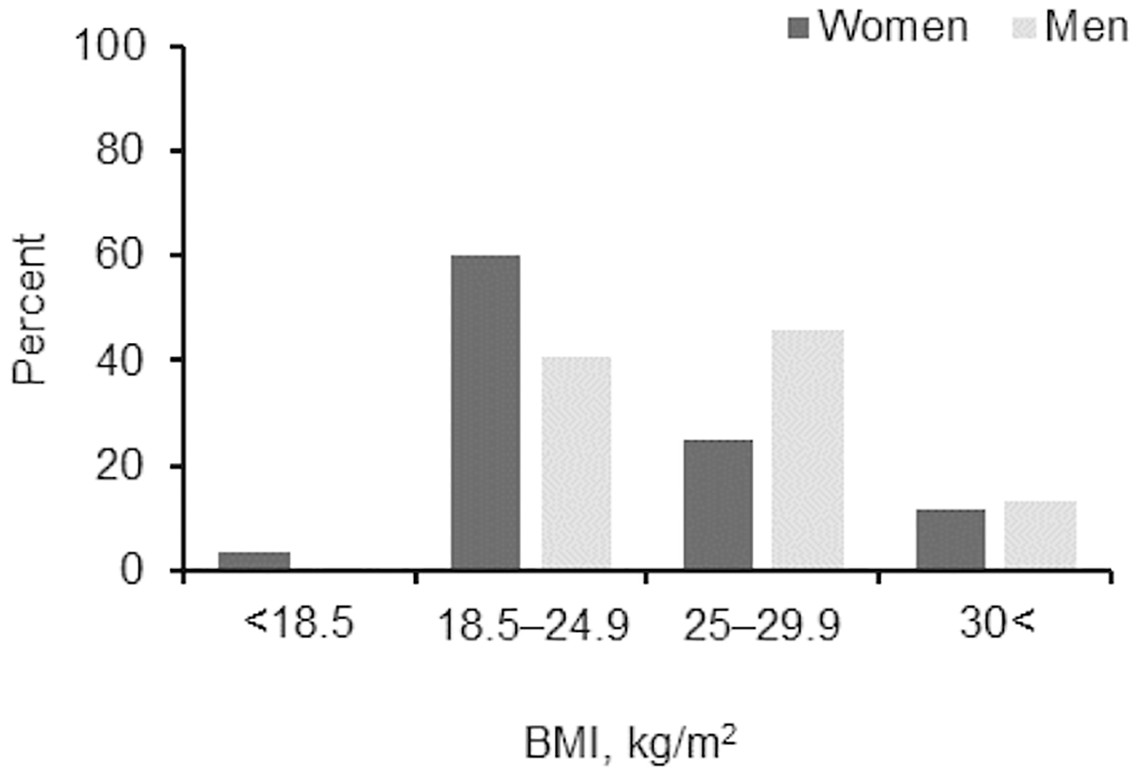
BMI structure of women and men.

There were 79.2% and 74.7% of women and men with university education, respectively. Overall, 30.9% of men and 17.5% of women did not exercise, while 1.8% of women and 5.8% of men were professional athletes. The subjective health rating of good and excellent was 57.3% and 15.5% for women, 55% and 24.9% for men (men vs women in excellent health, p < 0.001). There were 81.8% and 88.8% of women and men living in the city, respectively, while 79.2% of women and 71.2% of men did sitting and standing work. Descriptive data are presented in Tables 1 and 2. The age of all study groups was similar and there were more than 80% of men and women with a high university education. Women’s BMI was lower than men’s (p < 0.001). Among men, there were more people engaged in sports activities (p < 0.001) and their total MVPA was higher (p < 0.0001). Furthermore, men’s MVPA was higher both during work (p < 0.015) and during leisure time (p < 0.001). There were more women with normal body weight (p < 0.0001), and more men who were overweight (p < 0.0001) (Fig 1). Men’s SBP was significantly higher and morbidity was lower than women’s, but men and women did not differ according to subjective health assessment, sleeping time and frequency of overeating (Table 1). Interestingly, although men and women did not differ according to the subjective assessment of happiness, men’s vigour was significantly higher than women’s (p = 0.002) (Table 2). According to the logical thinking results, men did not differ from women, but women’s EI was higher than men’s (p < 0.001). Additionally, women’s perceived stress was higher than men’s (p < 0.001).

**Table 1:** Average (σ) descriptive data.

Parameter	Gender		p
	Female (n = 831)	Male (n = 309)	
**Age, years**	41.9 (11.6)	40.1 (11.2)	0.12
**BMI, kg/m** ^**2**^	24.2 (4.5)	26.5 (4.9)	0.001
**University-educated, %**	79.2	74.7	>0.05
**Untrained, %**	30.9	17.5	0.001
**Urban population, %**	82.8	84.8	>0.05
**MVPAw, min/week**	325.8 (98.4)	438.4 (126.4)	0.015
**MVPAh, min/week**	286.9 (101.1)	259.8 (88.9)	0.49
**MVPAlt, min/week**	174.2 (71.2)	358.4 (88.6)	0.0001
**MVPAtotal, min/week**	794.8 (189.4)	1148.9 (203.5)	0.0001
**Subjective health, points**	2.86 (0.69)	2.95 (0.71)	0.126
**Morbidity, %**	20.6	13.6	<0.05
**SBP, mm/Hg**	117.5 (11.2)	126.9 (9.4)	<0.0001
**Sleeping time, h**	7.34 (0.94)	7.28 (0.86)	0.37
**Overeating, %**	20.6	19.6	>0.05

**Note**. BMI–body mass index; MVPAh–household moderate to vigorous physical activity; MVPAlt–leisure-time moderate to vigorous physical activity; MVPAw–work-related moderate to vigorous physical activity; p–the level of marginal significance within a statistical hypothesis test; SBP–systolic blood pressure; σ–the standard deviation.

**Table 2:** Average (σ) data for happiness, vigour, logical thinking, emotional intelligence and perceived stress in women and men.

Parameter	Gender		p
	Female (n = 831)	Male (n = 309)	
**Happiness**	7.99 (1.3)	8.02 (1.41)	0.86
**Vigour**	8.96 (3.7)	9.95 (3.1)	0.002
**Logical thinking**	2.21 (0.51)	2.27 (0.45)	0.374
**Emotional intelligence**	128.3 (16.1)	122.1 (14.1)	<0.001
**Perceived stress**	16.8 (4.2)	14.2 (6.4)	<0.001

**Note.** p–the level of marginal significance within a statistical hypothesis test; σ–the standard deviation.

**Fig 2:**
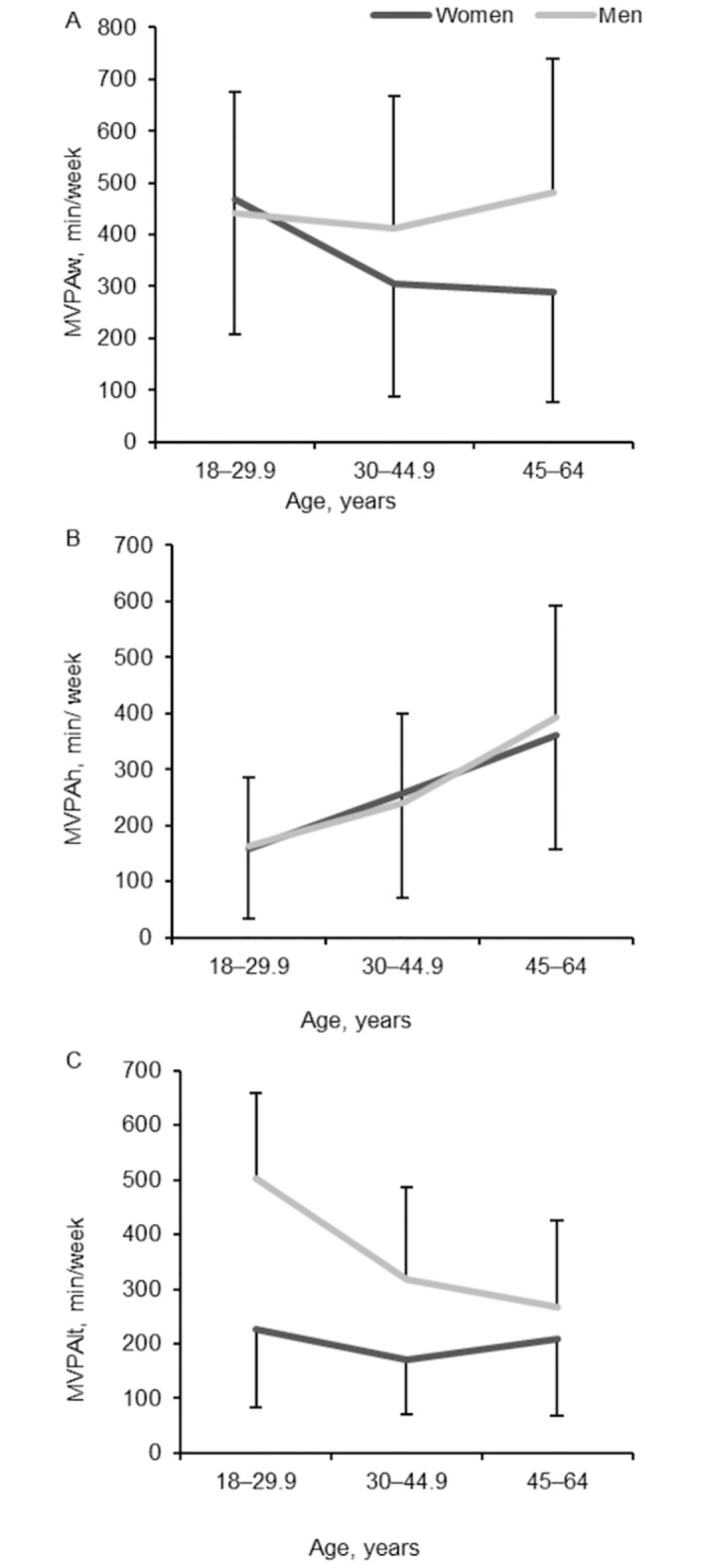
Age-dependent variation of work-related MVPA (A), household MVPA (B) and leisure-time MVPA (C) in women and men.

Two-way ANOVA revealed that work-related MVPA did not depend on age (p = 0.301) or gender (p = 0.068) (interaction of age x gender factors was not significant), but one-way ANOVA showed that work-related MVPA in women decreased with age (p = 0.021) (Fig 2). Household MVPA depended on age (p < 0.001) but did not depend on gender (p = 0.85) (interaction of age x gender factors was not significant). Interestingly, leisure-time MVPA depended on both gender (p < 0.001) and age (p = 0.005) (interaction of age x gender factors was significant, p = 0.041). However, one-way ANOVA revealed that leisure-time MVPA did not change with age in women (p = 0.238), while it changed (decreased) in men (p = 0.009).

The results of the regression analysis revealed that the PA paradox (i.e. the indicators were significantly correlated only with leisure-time MVPA) occurred in men and women in terms of health, happiness, vigour, and perceived stress, and only in women according to morbidity and overeating (Table 3). The paradox did not appear for both men and women, neither according to SBP, nor BMI, nor sleeping time, nor logical thinking, nor EI. It is interesting that for women all indicators except happiness were significantly correlated with age, for men education, morbidity, SBP, BMI, sleeping time, logical thinking and perceived stress were significantly correlated with age (while health, happiness, vigour, overeating and EI were not). No significant correlation was noted between BMI, logical thinking, sleeping time and PA. It is interesting that only women showed a significant correlation between PA and EI. For men there was a significant correlation between SBP and work-related MVPA.

**Table 3:** Correlation between physical activity structure (work-related MVPA, household MVPA, leisure-time MVPA) and health-related indicators.

Parameter		Gender							
		Female (n = 831)				Male (n = 309)			
		Age	MVPAw	MVPAh	MVPAlt	Age	MVPAw	MVPAh	MVPAlt
**Education**	**β**	**0.299**	**-0.105**	0.026	0.017	**0.27**	-0.085	-0.056	-0.053
	**p**	**<0.001**	**0.003**	0.46	0.62	**<0.001**	0.14	0.34	0.35
**Subjective health**	**β**	**-0.076**	0.05	-0.058	**0.135**	0.064	0.027	0.019	**0.147**
	**p**	**0.03**	0.168	0.112	**<0.001**	0.27	0.64	0.75	**0.011**
**Happiness**	**β**	0.051	0.036	0.019	**0.084**	0.094	0.11	0.007	**0.154**
	**p**	0.146	0.33	0.61	**0.018**	0.11	0.062	0.94	**0.01**
**Vigour**	**β**	**0.107**	0.021	0.055	**0.169**	0.04	0.001	0.033	**0.299**
	**p**	**0.002**	0.56	0.13	**<0.001**	0.49	0.99	0.058	**<0.001**
**Morbidity**	**β**	**0.233**	-0.062	0.023	**-0.078**	**0.223**	-0.034	0.014	-0.004
	**p**	**<0.001**	0.081	0.52	**0.021**	**<0.001**	0.56	0.82	0.84
**SBP**	**β**	**0.24**	**0.095**	-0.009	0.025	**0.135**	0.005	0.015	0.003
	**p**	**<0.001**	**0.017**	0.84	0.54	**0.039**	0.95	0.84	0.97
**BMI**	**β**	**0.27**	0.042	0.041	-0.064	**0.135**	0.049	0.09	-0.095
	**p**	**<0.001**	0.25	0.24	0.062	**0.021**	0.41	0.135	0.107
**Overeating**	**β**	**-0.087**	0.033	-0.057	**-0.097**	-0.051	-0.081	-0.019	-0.02
	**p**	**0.013**	0.36	0.12	**0.007**	0.39	0.18	0.76	0.73
**Sleeping time**	**β**	**-0.186**	-0.031	0.032	0.021	**-0.228**	-0.106	0.014	0.089
	**p**	**<0.001**	0.39	0.38	0.55	**<0.001**	0.066	0.82	0.127
**Logical thinking**	**β**	**-0.22**	-0.023	-0.054	0.006	**-0.33**	-0.063	0.054	-0.081
	**p**	**<0.001**	0.53	0.14	0.88	**<0.001**	0.28	0.36	0.11
**Emotional intelligence**	**β**	**0.119**	**0.108**	**0.111**	**0.082**	0.072	0.11	0.034	-0.077
	**p**	**0.001**	**0.003**	**0.002**	**0.018**	0.23	0.06	0.58	0.196
**Perceived stress**	**β**	**-0.27**	-0.055	-0.01	**-0.088**	**-0.123**	-0.092	0.013	**-0.179**
	**p**	**<0.001**	0.163	0.77	**0.009**	**<0.001**	0.075	0.85	**0.002**

**Note**. BMI–body mass index; MVPAh–household moderate to vigorous physical activity; MVPAlt–leisure-time moderate to vigorous physical activity; MVPAw–work-related moderate to vigorous physical activity; p–the level of marginal significance within a statistical hypothesis test; SBP–systolic blood pressure; σ–the standard deviation.

## Discussion

To our knowledge, this is the first study that has shown that the PA paradox is manifested (both in men and women, regardless of age) in subjective health, happiness, vigour and perceived stress. Thus, men’s and women’s subjective health, happiness and vigour were significantly positively, and perceived stress negatively correlated to leisure-time MVPA. This coincides with the findings of other researchers that not work-related PA, but leisure-time PA improves people’s health [20, 21, 22, 24, 25]. Our previous studies showed that mood profile depends on PA, but we did not distinguish the structure of PA in those studies, i.e. neither when it was applied nor at what intensity [34, 35].

Interestingly, our research showed that men and women with higher perceived stress scores had lower leisure-time MVPA. It is difficult to answer the question why people with a lower perceived stress level ‘choose’ healthier MVPA, i.e. leisure-time MVPA. Yan et al. showed that there is a direct relationship between perceived stress and emotional distress. It was established that during the COVID-19 pandemic, individuals who ‘adopted positive coping strategies suffered fewer symptoms of depression, compulsion-anxiety, and neurasthenia under stress, while negative coping strategies aggravated emotional distress’ [39]. Therefore, we can assume that leisure-time PA can be considered one of the positive coping strategies that can reduce boredom proneness, as the period of our research coincided with the end of a second wave of the COVID-19 pandemic in Lithuania.

It is interesting that, in our case, a PA paradox occurs only in women for morbidity and overeating. This partially coincides with the data of other researchers that leisure-time PA is associated with long-term sickness absence [37]. Studies conducted in China with a large sample showed that work-related PA was not associated with mortality risk [28]. However, in China, this PA paradox worked for both women and men. In our case, the PA paradox did not appear for both men and women, neither according to SP, nor BMI, nor sleeping time, nor logical thinking, nor EI. The Copenhagen City Heart Study showed that leisure-time PA had an impact on lower systolic SBP while occupational PA was associated with higher SBP [25]. However, we did not find a positive correlation between SBP and leisure-time MVPA in either men or women. Furthermore, our research showed a significant positive correlation between SBP and work-related MVPA in men. Therefore, it can be said that in our case, for men, ‘half’ of the PA paradox appeared for SBP (to be a ‘full’ paradox, if there was a significant correlation between leisure-time MVPA and SBP). Quite unexpectedly, we did not find significant correlation between leisure-time MVPA and EI and effectiveness of logical thinking. We especially expected such a correlation between leisure-time MVPA and EI, because our previous studies clearly showed that there is a significant positive correlation between PA and EI [6, 7]. Thus, our study did not confirm the second hypothesis that leisure-time MVPA should be significantly correlated with effectiveness of logical thinking and EI. Our current research shows that BMI, logical thinking and sleeping time were not significantly correlated with any form of MVPA. However, only women showed a significant correlation between all forms of MVPA and EI.

It is now becoming increasingly clear to researchers that the PA paradox is influenced by multiple and interrelated factors that are often difficult to examine in isolation. For example, Holtermann et al. presented six reasons why occupational PA does not improve cardiovascular health, while leisure-time PA does [40]. One of the most obvious reasons for this ‘physical activity paradox’ is that to strengthen health, it is more necessary to be physically active, followed by rest (as in leisure time, but not sitting for a long time). Additionally, if a lot of physical work is done throughout the workday, then it leaves less time for rest. For example, we found a significant negative correlation between education and work-related MVPA. This is quite easy to understand, as the more educated people are, the less their work is associated with high-intensity PA. However, in our case, we did not find significant correlation between education and leisure-time MVPA. Our research showed that leisure-time MVPA significantly depended on gender (men’s leisure-time MVPA was higher). Furthermore, leisure-time MVPA was especially high in young men (with age, leisure-time MVPA decreased for them, but did not change in women).

### Limitations

The main limitation of our research is that it does not allow us to determine the exact causal relationship between the structure of PA and health, well-being and healthy behaviour related indicators. The second limitation is that we assessed MVPA and other indicators (e.g. BMI, SBP, sleeping) based on questionnaires distributed online, so it would be more accurate to measure them objectively. The third limitation is that we chose only MVPA and did not analyse light PA such as walking or sedentary time, which could also affect health and health-related indicators, such as EI, perceived stress and effectiveness of logical thinking.

## Conclusions

Taken together, women’s and men’s subjective health, happiness and vigour were significantly positively, and perceived stress negatively correlated (regardless of age) only with leisure-time MVPA (and there was no significant correlation with work-related MVPA and household MVPA). For women (regardless of age), we also found that only leisure-time MVPA was significantly (inversely) correlated with morbidity and overeating rate. However, this PA paradox did not occur in either men or women for SBP, BMI, sleeping time, effectiveness of logical thinking and EI. ‘Healthy’, i.e. the amount of leisure-time PA in men decreases with age, while it does not change in women. We believe that this study has expanded a clearer understanding of the PA paradox, its possible application to improving the health of people in various age groups and raised new questions and new doubts.

## Supporting information

S1 Checklist (DOCX)

S1 Dataset (XLSX)
